# Hydrolysable chestnut tannins for reduction of postweaning diarrhea: Efficacy on an experimental ETEC F4 model

**DOI:** 10.1371/journal.pone.0197878

**Published:** 2018-05-25

**Authors:** Marion Girard, Sophie Thanner, Nicolas Pradervand, Dou Hu, Catherine Ollagnier, Giuseppe Bee

**Affiliations:** 1 Institute of Livestock Science, Agroscope, Posieux, Switzerland; 2 Institute of Agricultural Sciences, ETH Zürich, Zürich, Switzerland; INIA, SPAIN

## Abstract

An experimental model for postweaning diarrhea with enterotoxigenic *Escherichia coli* F4 (ETEC F4) was set up in piglets, and the efficacy of 1% chestnut-tannin extract in preventing diarrhea was subsequently assessed. In a first trial (infection model), 32 Swiss Large White piglets (age: 24 days; average BW: 7.8 ± 0.8 kg) were randomly assigned to two experimental groups (infected [INF], noninfected [NINF]). In a subsequent trial, 72 Swiss Large White piglets (age: 26 days; average BW: 7.4 ± 1.5 kg) were blocked by BW and assigned within block to four experimental groups: NINF-CO: not infected and fed a standard control starter diet (CO); INF-CO: infected and fed the CO diet; NINF-TA: not infected and fed the CO diet supplemented with 1% chestnut extract containing 54% of hydrolysable tannins (TA); and INF-TA: infected and fed the TA diet. Both diets (TA and CO) were formulated to be isocaloric and isoproteic and to meet or surpass the nutritional requirements. In both trials, four days after weaning, piglets assigned to the INF group received an oral suspension of ETEC F4. Fecal score, ETEC shedding in feces (only in trial 2), and growth performance traits were measured for the following 14 days post infection. In both trials, more than 50% of the INF piglets developed diarrhea within six days post infection. Tannins reduced (*P* < 0.05) the average fecal score, the percentage of piglets in diarrhea, and the duration of diarrhea, whereas feed intake and the average daily gain were unaffected.

## Introduction

Antimicrobials (AM) have revolutionized medicine in many respects, but they have also led to the rapid appearance of resistant mechanisms [1]. The prevalence of resistant microbes increases with the selection pressure exerted by AM used in human and veterinary medicine. The majority of AM (weight based) is used in agriculture [2]. Prophylactic and metaphylactic measures, consisting of treating entire groups that include both healthy and diseased animals, drastically enhance microbes’ exposure to AM. While targeting pathogens, AM also affect natural microflora, and especially the gut microflora. Selection and dissemination of antimicrobial-resistant microbes or plasmids frequently occur in the gastrointestinal tract of livestock. In addition, up to 90% of AM administered orally retain their antimicrobial activity in feces and potentially alter the soil microbial ecosystem after application of manure [3]. It has been suggested that the use of AM in livestock may increase the prevalence of resistant microbes in the human gastrointestinal tract [4] and may therefore increase the risk of human infections with resistant pathogens.

One of the production diseases for which AM are commonly used in pig production is postweaning diarrhea (PWD). Numerous stress factors are associated with weaning, including social, environmental, and dietary changes. These stressors can alter the homeostasis of intestinal microflora [5], rendering young piglets more inclined to gastrointestinal tract infections. The etiology of PWD is multifactorial, although it is commonly associated with the proliferation of beta-hemolytic enterotoxigenic *Escherichia coli* (ETEC), sometimes in association with rotavirus infections [5]. Fimbriated ETEC adhere to enterocyte-specific receptors and secrete enterotoxins, heat-labile toxin (LT), and heat-stable toxin (ST), causing electrolyte and net fluid losses [6]. This results in dehydration, weight loss for the piglet, and sometimes death [7]. Thus, managing the period around weaning is challenging for the piglet and the farmer. With the increasing occurrence of antimicrobial resistance, there is an urgent need to reduce AM use by identifying dietary alternatives for alleviating PWD in pig production [8–12]. Polyphenols, such as tannins from oak (*Cortex quercus*), were used to treat diarrheal disease in the preantibiotic era [13]. Plant polyphenols are known to have antimicrobial properties [14] and inhibitory effects on bacterial toxins [15]. However, interactions with bacterial toxins seems to be specific [16], as only a few tannins are able to reduce ETEC diarrhea [17]. Chestnut-tannin extract may be a good candidate for decreasing PWD, because it already possesses in vitro bactericidal activity on several bacteria [18, 19]. The main objective of this study was to assess the effect of hydrolysable chestnut-tannin extract on the prevalence of diarrhea using an ETEC-infection model with weaned piglets.

## Materials and methods

All experimental procedures were in compliance with Swiss animal welfare guidelines and were approved (No. 2014_54_FR) by the Cantonal Veterinary Office of Fribourg (Switzerland). This study was performed at the piggery of the research station Agroscope–Posieux (Switzerland).

### Harboring the infectious ETEC strain

The native ETEC strain used in this study was isolated from a weaned piglet at the piggery of the research station Agroscope–Posieux (Switzerland); the piglet exhibited acute PWD. The strain was resistant to sulfamethoxazole (smx^R^) and harbored the F4 fimbriae gene (K88ac+ subvariant), the heat-labile toxin gene (LT+), and the heat-stable toxin gene (STb+) but not the STa gene, as determined by polymerase chain reaction (PCR; primers are listed in Table 1 [20–22]). In order to obtain a convenient selection marker to retrieve the ETEC strain from feces at the output of the infection model, a spontaneous mutant resistant to rifampicin was searched for and isolated. To do so, the strain was cultured overnight in Luria-Bertani broth (Becton Dickinson, UK) at 37°C with 180 revolutions per minute (rpm) in a shaker incubator, and 100 μl were transferred on several Eosin-Methylene Blue (EMB) agar plates (Oxoid CM0069, UK) supplemented with 50 μg/ml rifampicin (rif50). A colony growing on one of these plates was isolated, purified, and checked again by PCR for the presence of K88ac, LT toxin, and STb genes. This strain would serve as the ETEC used in the infection model described below.

**Table 1:** Primers used to characterize the enterotoxigenic *Escherichia coli* F4 strain.

Primer name	Sequence (5’-3’)	Reference
K88ac Fwd	TTTGCTACGCCAGTAACTG	[20]
K88ac Rev	TTTCCCTGTAAGAACCTGC	
K88ab Fwd	TTGCTACGCCAGTAAGTGGT	
K88ab Rev	CGAAACAGTCGTCGTCAAA	
K88ad Fwd	GGCACTAAAGTTGGTTCA	
K88ad Rev	CACCCTTGAGTTCAGAATT	
LT Fwd	TAGAGACCGGTATTACAGAAATCTGA	[21]
LT Rev	TCATCCCGAATTCTGTTATATATGTC	
STa Fwd	TCTTTCCCCTCTTTAGTCAG	[22]
STa Rev	ACAGGCCGGATTACAACAAAG	
STb Fwd	GCCTATGCATCTACACAATC	
STb Rev	TGAGAAATGGACAATGTCCG	
LT qPCR Fwd	GGCGTTACTATCCTCTCTAT	[23]
LT qPCR Rev	TGGTCTCGGTCAGATATGT	

Inocula were prepared by growing the strain overnight in sterile Luria-Bertani broth, as described above. The culture was centrifuged for 10 min at 6000 rpm in order to get rid of the toxin-laden supernatant. The bacterial pellet was then resuspended in 1x phosphate buffered saline (PBS) and adjusted to a final concentration corresponding to 10^8^ CFU/ml (using the optical density at 600 nm absorbance, Biowave II WPA, LABGENE Scientific SA, Châtel-Saint-Denis, Switzerland).

### Animals and rearing conditions

Two weeks before the expected day of farrowing, sows were vaccinated with Porcilis® Porcoli DF (ad us. vet., MSD Animal Health GmbH, Lucerne, Switzerland), a suspension for injection that contains deactivated fimbria adhesins of *E*. *coli* F4ab, F4ac, F5, F6, and the LT-toxoid. Within seven days after birth, an ear sample 2 mm in diameter was taken from each piglet with special pliers, and its DNA was analyzed to determine whether the animal would be susceptible (i.e., harboring a genetic marker for F4ab/ac receptor) or resistant to ETEC F4 infection (i.e., harboring a genetic variant of the marker for F4ac/ab receptor that makes piglets resistant) [24].

In both trials, piglets were reared in pairs in individual pens (1.6-m^2^ concrete floor and 1-m^2^ slatted metal floor) and had access to a wooden box with straw placed underneath a heating lamp. Each pen was equipped with nipple drinkers, giving ad libitum access to clean, fresh water. An electrolyte (NaCl hypertonic) solution was also available for drinking. From weaning to 18 days post weaning (which marked the end of the two trials), ambient temperature was maintained at around 28°C.

### Experimental designs

Trial 1: To set up and validate the ETEC infection model of PWD, 32 Large White piglets ([average ± standard deviation] 24 ± 1 days of age and 7.8 ± 0.8 kg BW) were included. On the day of weaning (day -4), they were blocked within littermates by BW and assigned equally to either the infected (INF) or noninfected (NINF) group. Directly at weaning, piglets had ad libitum access to a standard starter diet formulated to meet or surpass the nutritional requirements (Table 2). Four days postweaning (day 0), piglets were offered 5 ml of the previously described ETEC suspension containing 10^8^ CFU/ml (INF) or 5 ml of PBS (NINF) by oral administration with a syringe. Of these 32 piglets, two INF and one NINF were determined to be resistant to ETEC F4.

**Table 2:** Ingredient composition and chemical content (g/kg) of the experimental diets^a^.

Ingredient	CO	TA
Barley, ground	33.39	33.39
Oat flakes	2.00	2.00
Corn, ground	20.00	20.00
Wheat, ground	9.20	9.20
Wheat meal	0.38	0.38
Whey permeate	5.00	5.00
Rapeseed oil	3.36	3.36
Potato protein	6.47	6.47
Soybean meal	9.88	9.88
Wheat bran	2.42	1.42
Apple pomace, dried	4.00	4.00
L-lysine-HCl (79%)	0.40	0.40
L-threonine (99%)	0.01	0.01
Dicalcium phosphate	1.37	1.37
Sodium chloride	0.20	0.20
Calcium formate	1.00	1.00
Pellan^b^	0.30	0.30
Vitamin–mineral premix without Fe^c^	0.40	0.40
Luctarom^d^	0.01	0.01
Greencab-70-C^e^	0.20	0.20
Natuphos 5000 G^f^	0.01	0.01
HTE^g^	-	1.00
Nutrient and digestible energy content (expressed per kg as fed) composition, %		
Dry matter, g	889	890
Crude protein, g	166.7	164.3
Crude fat, g	55.9	51.9
Crude fiber, g	32.6	40.5
Fe, mg	100.6	100.6
DE, MJ^i^	14.0	13.9

^a^ CO = control diet; TA = control diet supplemented with 1% hydrolysable chestnut-tannin extract

^b^ Pellet binding aid: Pellan, Mikro-Technik, Bürgstadt, Germany

^c^ Supplied per kg of diet: vitamin A, 8000 IU; vitamin D_3_, 1000 IU; vitamin E, 25mg; menadione, 3 mg; thiamine, 2 mg; riboflavin, 5 mg; biotin, 0.1 mg; niacin, 20 mg; pantothenic acid, 15 mg; iodine, 0.15 mg as calcium iodate; copper, 6 mg as copper sulfate; manganese, 10 mg as manganese oxide; zinc, 75 mg as zinc oxide; selenium, 0.2 mg as sodium selenite

^d^ Luctarom, Lucta, Montornès del Vallès, Spain

^e^ Coated calcium butyrate: Greencab 70-c, Brenntag, Denmark

^f^ Phytase supplemented at 500 units of aspergillus niger phytase/kg diet

^g^ Hydrolysable chestnut-tannin extract (Silvafeed Nutri P/ENC for Swine, Silvateam, Italy)

^i^ DE = digestible energy content estimated according to the Swiss (Agroscope, 2017) database, taking into account the relative amount of each feed ingredient in the diet.

Trial 2: The objective of trial 2 was to assess the effects of dietary hydrolysable tannin supplementation on the incidence of PWD in INF and NINF piglets. Piglets were weaned at 26 ± 2 days of age with a weaning weight of 7.4 ± 1.5 kg BW. The trial was arranged according to a 2 × 2 factorial design with two levels of infection (INF vs. NINF) and two diets (unsupplemented control starter diet [CO; Table 1] and the CO diet supplemented with 1% of chestnut-tannin extract [TA; Table 1]), resulting in four experimental groups (INF-CO, NINF-CO, INF-TA, NINF-TA) of 18 piglets each. The two diets were formulated according to current Swiss recommendations for pigs [25]. The commercial hydrolysable chestnut-tannin extract (HTE; Silvafeed Nutri P/ENC for Swine, Silvateam, Italy) used in the trial contained 45% gallotannins, 9% ellagitannins, and 3.7% gallic acid. Rearing condition, infection procedure, and feed access were equal to those used in trial 1. Three INF-CO, 2 INF-TA, 1 NINF-CO, and 1 NINF-TA (out of 72 piglets) were genotyped as resistant to ETEC F4.

### Clinical parameters and laboratory analyses

From the day of infection (day 0, i.e., four days after weaning) to 14 days post infection, fecal scores were regularly assessed using the score scheme previously proposed [26]: 1 = dry, pelleted feces; 2 = molded feces; 3 = moist, cow-dung appearance; 4 = diarrhea; 5 = watery diarrhea. Piglets were considered to have diarrhea when the fecal score was 4 or above. Individual bodyweight and feed intake per pen were determined weekly and daily, respectively. General health status was monitored daily throughout the trial.

In trial 2, fecal samples were collected directly from the rectum on day 0 (before infection) and day 4. These samples were used to determine the presence of the ETEC strain applied with the suspension. The presence of the ETEC strain was detected by quantitative real-time PCR (qPCR). The DNA of the dried feces sample was extracted using QIAamp® Fast DNA stool Mini Kit (Qiagen GmbH) according to the manufacturer’s instructions. The qPCR test was performed using primers targeting the heat-labile (LT) toxin gene (Table 2, [23]). A Bio-Rad CFX96 Touch PCR and a KAPA SYBR® FAST qPCR universal kit was used (Kapabiosystem, USA). The DNA of the infective ETEC F4ac strain was used as the standard curve. The DNA concentration of standard 1 was 3.1 ng/μl, and serial 1:10 dilutions were performed for standards 2 to 7. A total of 15 ng DNA of each feces sample was used for qPCR. Thermal cycling conditions were 95°C for 3 min followed by 40 cycles at 95°C for 10 s, 58°C for 30 s, and 72°C for 30 s. Melting curve analysis confirmed primer specificities with the following thermal cycling conditions: 95°C for 10 s and increments of 0.5°C per 5 s from 65 to 95°C. In addition, in trial 2, when a piglet developed watery diarrhea (i.e., a fecal score of 5), an additional rectal swab sample was collected to check for the presence of other bacterial and viral pathogens causing PWD. Analyses were performed by a commercial laboratory (Idexx Diavet Labor AG, 8806 Bäch SZ, Switzerland).

### Data analysis and statistics

In trial 1, data for growth performances, days in diarrhea, and fecal score were analyzed using PROC MIXED of SAS (version 9.2, SAS Institute Inc., Cary, NC, USA), where the effect of infection, run, and time were considered fixed effects and the piglet and litter random effects. The percentage of piglets with diarrhea, following a binomial distribution, was analyzed with the GLIMMIX procedure of SAS (version 9.2, SAS Institute Inc., Cary, NC, USA). In addition, data for fecal score and percentage of piglets with diarrhea were analyzed using the repeated statement.

In trial 2, data for feed intake per pen (FI), weight of the piglets, average daily gain (ADG), and feed efficiency (per pen) were analyzed with linear mixed models using R software (R Core Team, 2016). Discrete dependent variables were modeled using R: counts for days in diarrhea as quasi-Poisson, ordinal fecal scores as proportional odds logistic regression using generalized estimating equations, and dichotomous responses for percentage of diarrhea as binary generalized linear mixed model. The initial models included the effects of infection, feed, run, time (weeks or days), genotype (susceptible or resistant), and sex and the first-order interactions infection × feed, infection × time, and feed × time as fixed effects, and they included pairs of piglets (for feed intake) or piglets and litter as random effects. In general, the models were reduced by stepwise exclusion of nonsignificant interactions and factors (except feed and infection) on a P-level of 0.10. Least-squares means of the response variables and Tukey-Kramer pairwise comparisons were computed, and differences were considered significant if *P* < 0.05 and considered a tendency if *P* ≤ 0.10. Because of the non-normality of the data, ETEC shedding was converted in log_10_ (1+N) and analyzed with a nonparametric Wilcoxon test with the NPAR1WAY procedure of SAS (version 9.2, SAS Institute Inc., Cary, NC, USA).

## Results

### Trial 1

**Fig 1:**
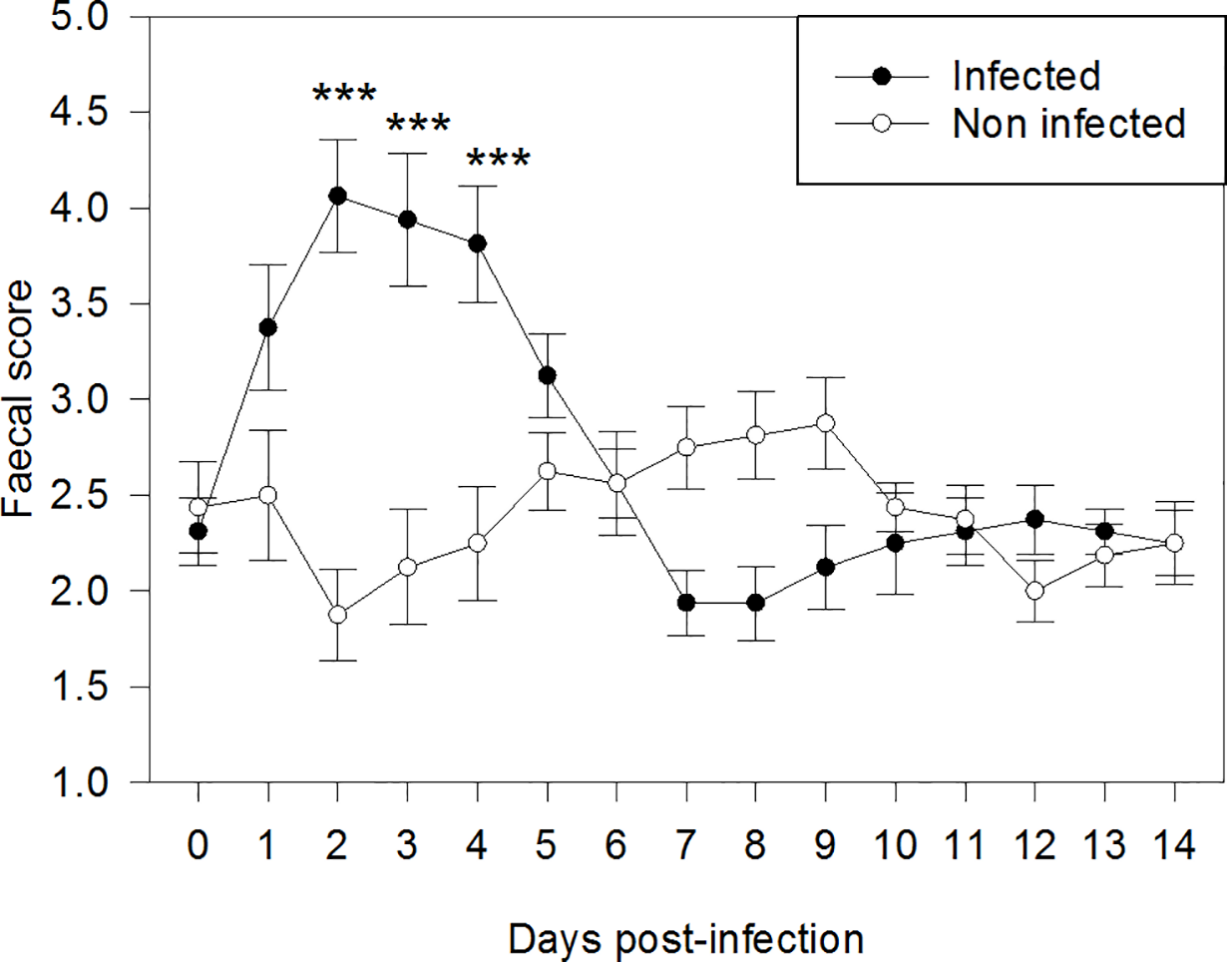
Average fecal score (± standard error) of infected and noninfected piglets monitored during 14 d post infection. *P*-values for the main factors: infection: *P* < 0.001; days: *P* < 0.001; infection × days: *P* < 0.001. *** indicates differences between infected and noninfected piglets at *P* < 0.001 within the same day. Four days post weaning (day 0), infected piglets were orally administered 5 ml of the ETEC F4 suspension containing 10^8^ CFU/ml, whereas NINF piglets received 5 ml of PBS orally.

**Fig 2:**
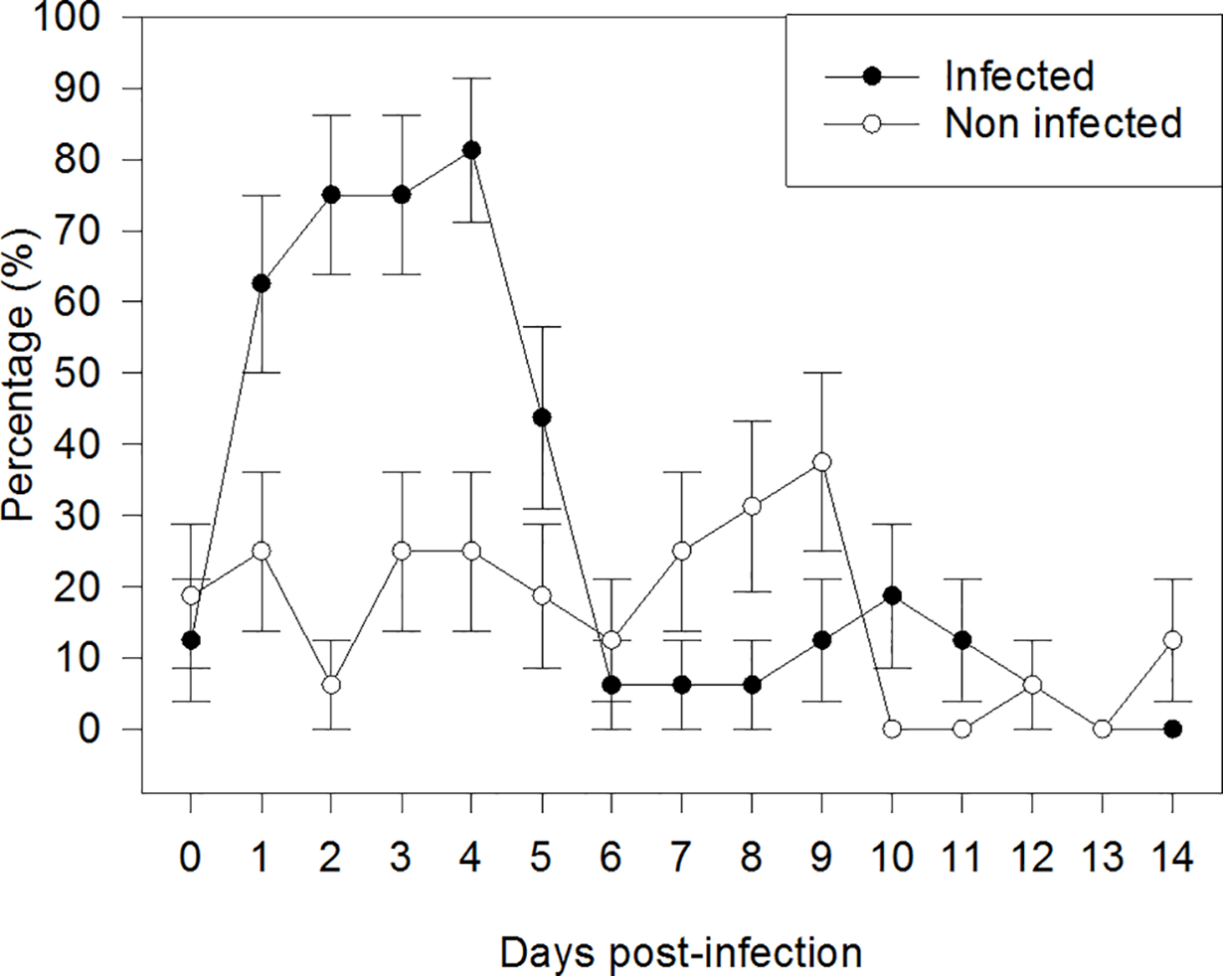
Percentage of piglets exhibiting diarrhea (i.e., fecal score ≥ 4) in the infected and noninfected groups (trial 1). *P*-values for the main factors: infection: *P* = 0.01; days: *P* = 0.42; infection × days: *P* = 0.07. At four days post weaning (day 0), infected piglets were orally administered 5 ml of the ETEC F4 suspension containing 10^8^ CFU/ml, whereas NINF piglets received 5 ml of PBS orally.

Age at weaning, BW at weaning and at day 38 of age, and consequently ADG did not differ (*P* > 0.10) between INF and NINF piglets (Table 3). Accordingly, no differences (*P* > 0.10) in average daily feed intake per pen were observed between INF and NINF piglets during experimental days 0–7 and 8–14. In the first week post infection, diarrhea lasted on average 2 days longer (*P* = 0.002) in INF piglets than in NINF piglets. The fecal score was greater (*P* < 0.001) in INF than in NINF piglets for the entire duration of the study (Fi 1). The impact of ETEC F4 infection was more evident in the first days after infection, when the fecal score increased markedly in INF piglets compared to NINF piglets, whereas it leveled out between the two treatments at the end of the 14-day trial (infection × time interaction: *P* < 0.001; Fig 1). The percentage of INF piglets developing diarrhea was significantly greater (*P* = 0.01; maximum 80% on day 4 post infection) than for NINF piglets (25% on the same day; Fig 2). There was a tendency of infection × time interaction (*P* = 0.07), due mainly to the greater development of diarrhea at days 2, 3, and 4 in INF piglets compared to NINF piglets. Interestingly, 10 of the 16 NINF piglets had diarrhea at least once in the second week (days 7–14 post infection) compared to 4 of the 16 INF piglets. No mortality occurred among the 32 piglets.

**Table 3:** Growth performance and days in diarrhea of infected (INF, *n* = 16) and noninfected piglets (NINF, *n* = 16)^a^.

	INF	NINF	SEM^b^	*P*-value
Age at weaning, d	24	24	0.1	1.00
BW at weaning, kg	7.85	7.74	0.198	0.71
BW 18 d after weaning (14 d post infection), kg	11.46	11.28	0.438	0.78
Average daily gain^c^, g/d	200	197	16.9	0.87
Days in diarrhea^d^	3.6	1.6	0.43	0.002

^a^ At four days post weaning, INF piglets were infected orally with 5 ml of the ETEC suspension containing 10^8^ CFU/ml (INF), whereas NINF piglets received 5 ml of PBS orally.

^b^ SEM = pooled standard error of mean.

^c^ Average daily gain was calculated over the period between weaning and 18 d after weaning.

^d^ Days in diarrhea (i.e., with fecal score ≥ 4) were determined in the first experimental week.

### Trial 2

**Fig 3:**
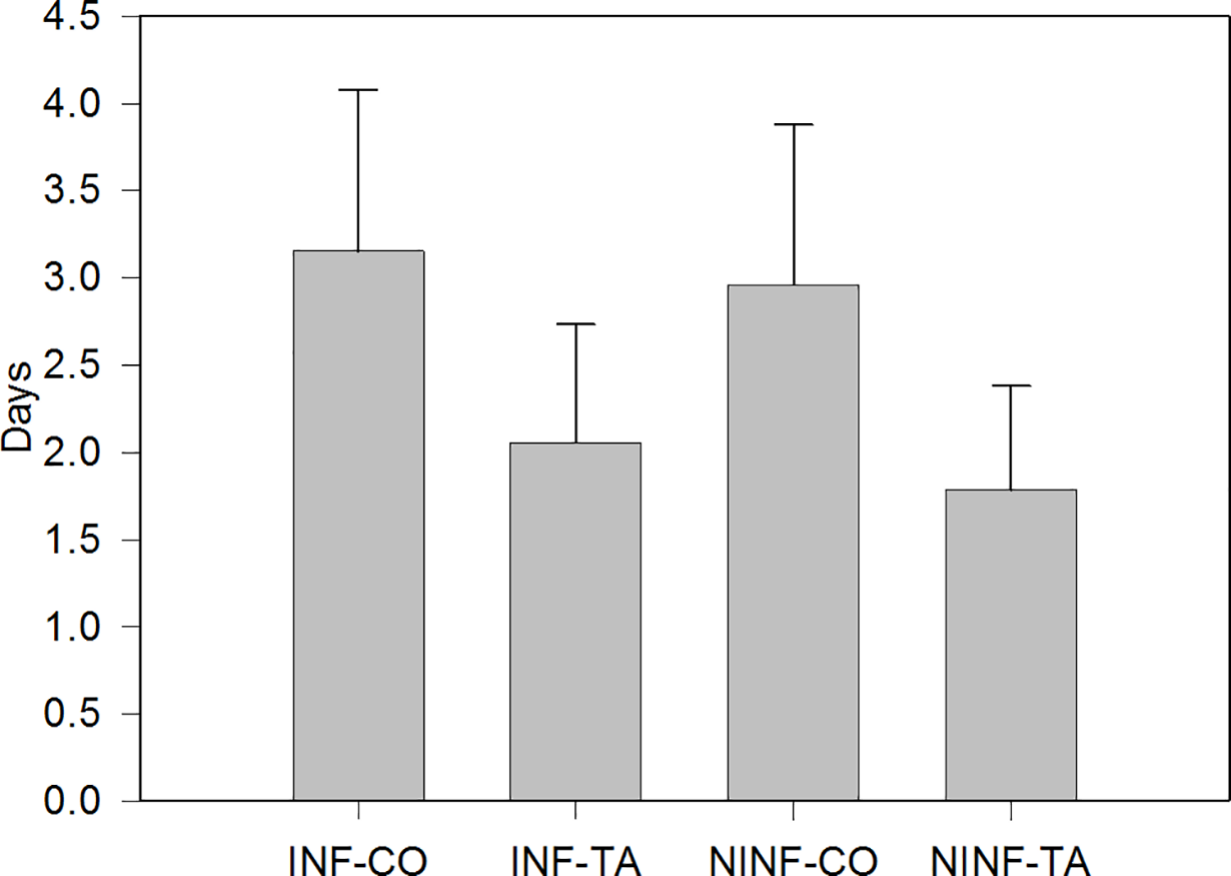
Number of days in diarrhea (i.e., fecal score ≥ 4) of piglets from the INF-CO, INF-TA, NINF-CO and NINF-TA groups. *P*-values for the main factors: infection: *P* = 0.67; diet: *P* = 0.008. Piglets of the INF-CO and INF-TA group were infected orally with 5 ml of the ETEC F4 suspension containing 10^8^ CFU/ml four days post weaning and fed either an unsupplemented standard control starter diet or a control standard diet supplemented with 1% chestnut-tannin extract from weaning (day -4) for 18 days, respectively. The piglets of the NINF-CO and NINF-TA group were fed the same diets for the same time span as previously described but received orally 5 ml of PBS.

**Fig 4:**
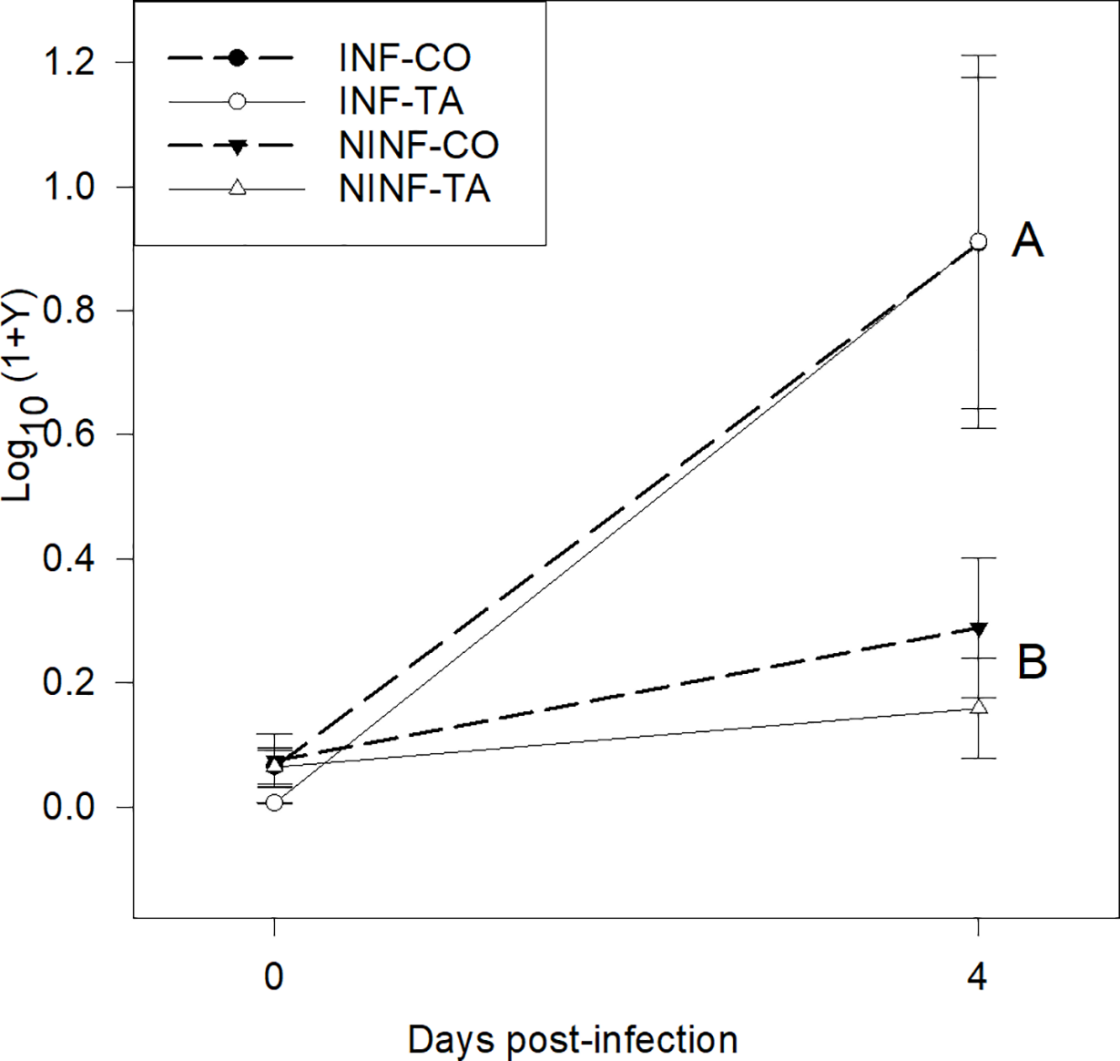
LT gene abundance determined by qPCR in the feces of INF-CO, INF-TA, NINF-CO, and NINF-TA piglets at days 0 and 4 post infection. Data are expressed as Log_10_ (1+Y), where Y represents the ng LT-DNA per g feces. *P*-values of the Wilcoxon test for the main factors at day 0: infection: *P* = 1.00; feed: *P* = 0.26; and at day 4: infection: *P* = 0.009; diet: *P* = 0.34. ^A,B^ indicates differences between infected and noninfected piglets at *P* = 0.009 on day 4. Piglets in the INF-CO and INF-TA groups were infected orally with 5 ml of the ETEC F4 suspension containing 10^8^ CFU/ml four days post weaning and fed either an unsupplemented standard control starter diet or a control standard diet supplemented with 1% chestnut-tannin extract from weaning (day -4) for 18 days, respectively. Piglets in the NINF-CO and NINF-TA groups were fed the same diets for the same time span as previously described but received 5 ml of PBS orally.

Over the entire course of the experiment, NINF piglets tended to be heavier (*P* = 0.08) than INF piglets (8.93 and 8.40 kg for NINF and INF piglets, respectively) (Table 4). Regardless of whether piglets received the ETEC F4 solution or PBS orally, dietary HTE supply had no effect (*P* > 0.10) on ADG, average daily FI, and feed efficiency. There was a significant infection × days interaction (*P* = 0.03) for ADG, which was probably due to a compensatory growth of the INF piglets who reached the same ADG as NINF piglets in the second week post infection. Surprisingly, infection had no effect (*P* = 0.19) on fecal score (Table 5). However, dietary HTE supplementation lowered (*P* < 0.001) the average fecal score monitored for 14 d in both INF and NINF piglets by approximately 0.5 units (2.58 vs. 3.07). Accordingly, the number of days in diarrhea was lower (*P* = 0.008) in TA (INF-TA and NINF-TA) than in CO piglets (INF-CO and NINF-CO; Fig 3) but was unaffected (*P* = 0.67) by the extent of infection to which piglets were subjected. Despite not being significant, it is noteworthy that from day 1 to 5 post infection, the percentage of piglets exhibiting diarrhea was lower in the NINF than in the INF group (*P* = 0.55; Table 6). There was a significant infection × days interaction (*P* = 0.01) due to the development of diarrhea in the NINF-CO group at day 6 and to a lesser extent at day 7. Over the 14-d postinfection period, the addition of HTE decreased (*P* = 0.007) by half the percentage of piglets with diarrhea (43% and 26% for CO and TA groups, respectively). Infection had no effect (*P* = 1.00) on the excreted quantity of ETEC F4 in the feces, as determined by qPCR at day 0 (i.e., before infection) (Fig 4). However, four days after infection, INF piglets excreted more ETEC F4 (*P* < 0.05) than did NINF piglets. On the other hand, feed had no effect (*P* > 0.10) on ETEC F4 excretion on days 0 and 4. Of the 32 swabs sampled for laboratory analyses (pathogen analyses), 20 contained rotaviruses (8 INF/12 NINF), and the main isolated bacteria was *E*. *coli*.

**Table 4:** Growth performance of infected (INF) and noninfected piglets (NINF) fed either a control standard starter diet or the control starter diet (CO) supplemented with 1% chestnut extract (TA)^a^^,^^b^.

Infection	NINF		INF		SEM	*P*-values^c^			
Diet^d^	CO	TA	CO	TA		I	D	W	I × W
BW (post infection), kg									
at D 0	7.35	7.80	7.57	7.69	0.521	0.08	0.49	< 0.001	0.57
at D 7	8.54	8.75	8.06	8.38					
at D 14	9.99	10.07	9.41	10.27					
Daily feed intake, g/d									
D 0*–*7	551	555	488	517	81.8	0.35	0.30	< 0.001	0.91
D 8*–*14	1001	1059	946	1043					
D 0*–*14	776	807	717	780	45.3	0.35	0.31	-	-
Average daily gain, g/d									
D 0*–*7	135	122	84	104	24.4	0.45	0.46	< 0.001	0.03
D 8*–*14	208	188	192	270					
D 0*–*14	171	155	138	187	20.9	0.97	0.44	-	-
Gain-to-feed, g/g									
D 0*–*7	0.51	0.42	0.37	0.42	0.077	0.89	0.92	0.86	0.18
D 8*–*14	0.43	0.36	0.44	0.53					
D 0*–*14	0.44	0.38	0.38	0.46	0.053	0.88	0.82	-	-

^a^ At four days post weaning, INF piglets were infected orally with 5 ml of the ETEC suspension containing 10^8^ CFU/ml (INF), whereas NINF piglets received 5 ml of PBS orally.

^b^ Results are presented as least square of means and pooled standard error of means (SEM).

^c^ *P*-values for the main factors infection (I), diet (D), W (week), and infection × week interaction (I × W).

^d^ The commercial chestnut-tannin extract (HTE; Silvafeed Nutri P/ENC for Swine, Silvateam, Italy) contained 45% gallotannins, 9% ellagitannins, and 3.7% gallic acid.

**Table 5:** Average fecal score of INF-CO, INF-TA, NINF-CO, and NINF-TA piglets^a^^,^^b^ from d 0–7 post infection (daily) and at d 14.

Infection	NINF		INF		Pooled SE	*P*-values^c^			
Diet	CO	TA	CO	TA		I	D	days	D × days
days									
0	2.7	1.7	2.3	2.1	0.12	0.19	< 0.001	< 0.001	< 0.001
1	3.2	2.3	3.7	3.2	0.17				
2	3.2	2.9	3.4	3.2	0.17				
3	2.9	2.7	3.7	3.2	0.18				
4	3.2	2.2	3.6	2.9	0.16				
5	2.9	2.3	3.1	2.8	0.11				
6	3.2	2.5	2.9	2.4	0.11				
7	2.8	2.7	2.4	2.2	0.12				
14	2.2	1.7	1.9	1.7	0.08				

^a^ At four days post weaning, INF-CO and INF-TA piglets were infected orally with 5 ml of the ETEC suspension containing 10^8^ CFU/ml (INF) and fed either an unsupplemented standard control starter diet or a control standard diet supplemented with 1% chestnut-tannin extract from weaning (day -4) for 18 days, respectively. Piglets in the NINF-CO and NINF-TA groups were fed the same diets for the same time span as previously described but received 5 ml of PBS orally.

^b^ Results are presented as means and pooled standard error (SE).

^c^ *P*-values for the main factors infection (I), diet (D), days, and diet × days interaction (D × days).

**Table 6:** Percentage of piglets in the INF-CO, INF-TA, NINF-CO, and NINF-TA groups exhibiting signs of diarrhea (i.e., fecal score ≥ 4)^a^^,^^b^.

Infection	NINF		INF		Pooled SEM	*P*-values^c^			
Diet	CO	TA	CO	TA		I	D	days	I × days
days									
0	28	6	17	11	4.3	0.55	0.007	< 0.001	0.01
1	44	28	67	33	5.9				
2	50	39	61	39	5.9				
3	44	44	56	44	5.9				
4	44	17	50	39	5.7				
5	33	11	44	28	5.4				
6	56	22	28	11	5.4				
7	39	28	22	17	5.2				
14	6	0	6	0	2				

^a^ At four days post weaning, INF-CO and INF-TA piglets were infected orally with 5 ml of the ETEC suspension containing 10^8^ CFU/ml (INF) and fed either an unsupplemented standard control starter diet or a control standard diet supplemented with 1% chestnut-tannin extract from weaning (day -4) for 18 days, respectively. Piglets in the NINF-CO and NINF-TA groups were fed the same diets for the same time span as previously described but received 5 ml of PBS orally.

^b^ Results are presented as least square of means and pooled standard error of means (SEM).

^c^ *P*-values for the main factors infection (I), diet (D), days, and infection × days interaction (I × days).

## Discussion

The ETEC F4 infectious strain was quite representative of the strains commonly found in PWD worldwide, as it harbored adhesin F4ac and toxins LT+ and STb+ [7]. Previous studies of artificial ETEC infection models reported an incidence of diarrhea ranging from 50% to 70% [26, 27] during the first two weeks post weaning. In the present study, greater occurrence of diarrhea was observed in INF piglets in trial 1, as prevalence was 60%–80% from day 1 to 4. Duration of diarrhea was also longer, with 3.6 days on average in the current study compared to 1.7 days in the study of Madec et al. [26]. The greater prevalence and duration of diarrhea were achieved while administering the lowest infectious dose of those proposed by Madec et al. [26]. This low infective dose allowed diarrhea to be induced, but it was not severe enough to impact growth performance traits, even if there was a tendency to decrease BW within two weeks post infection. Furthermore, INF piglets shed more ETEC F4 than did NINF piglets. The infectious inocula were administered only once, compared to administration of the inoculum for up to eight consecutive days in other studies [26, 28]. The timing of infection (i.e., four days after weaning) was chosen based on outcomes from other studies [26, 27] and in order to target the highest susceptibility windows after weaning.

The infectious model was repeatable, as the average fecal score, days in diarrhea, and percentage of piglets in diarrhea were within the same range in trials 1 and 2. The prevalence of diarrhea in NINF (trial 1), NINF-CO, and NINF-TA (trial 2) was unexpectedly great. This contrasts with the findings of the study of Madec et al. [26], in which none of the noninfected piglets developed diarrhea within the three weeks after weaning. However, the Madec study was performed in a high-disease-security experimental facility with Specific Pathogen Free piglets, not in an experimental and conventional piggery as in the current study. Furthermore, the ETEC infective strain used here was already part of the environmental flora of our piggery. In the present study, two processes may explain the relatively high incidence of diarrhea in NINF piglets: the presence of rotaviruses in the environment, as confirmed by the laboratory analyses performed in trial 2, and potential cross contamination between INF and NINF piglets. Cross contamination could explain the rise in diarrhea incidence observed in NINF piglets after day 6 in both trials. Although rotavirus is not regarded as a primary cause of PWD, it favors ETEC colonization by modifying the gut environment [29]. In their PWD induction protocol, Niewold et al. [30] inoculated rotaviruses prior to artificial infection with ETEC in weaned piglets.

For trials 1 and 2, a control starter diet with low crude protein and minimal iron supplementation was formulated to minimize the risk of PWD [31–33]. Iron is an essential nutrient for basic bacterial metabolic pathways, but also an essential mineral for mammals [34]. The diet was formulated to meet the minimum iron physiological requirements [33]. In trial 2, the 1% chestnut extract in the TA diet replaced 1% of wheat bran in the CO diet (as fiber source), knowing that the latter has been shown to reduce prevalence of PWD [35]. In the present study, the HTE supplement combined with the wheat bran exceeded the effect of the wheat bran alone, as all INF-TA and NINF-TA piglets had lower prevalence of diarrhea, lower fecal score, and fewer days in diarrhea compared to INF-CO and NINF-CO piglets.

Previous studies [17, 36] have evaluated the efficacy of polyphenols in preventing PWD caused by ETEC. Several mechanisms have been proposed to be involved in the antimicrobial property of polyphenols, including cell wall adherence, membrane integrity disturbance, and cell growth inhibition. One mode of action of the hypothesized polyphenols is linked to their capacity to bind proteins, implying that they could inactivate microbial adhesins, extracellular microbial enzymes, and envelope transport proteins [14]. Numerous polyphenols have a proven capacity to inhibit ETEC adhesion to intestinal epithelium [36, 37], and a few polyphenols are able to inactivate in vitro enterotoxins [15, 36]. Indeed, tannins are able to bind to a variety of substrates. For instance, hydrolysable tannins contained in *Terminalia chebula* fruits were able to bind to and inhibit the bacterial efflux pumps that are often involved in multidrug resistances [38]. Polyphenols may also deprive bacteria of essential substrates for growth [39]. By capturing iron, tannic acids [40] reduce iron absorption in laboratory rats and deprive iron-requiring enteric pathogens such as *E*. *coli* [41], thus reducing coliform fecal count. Tannins are often reported as antinutritional products because they may negatively affect protein digestion [42]. Tannic acids also seem to negatively impact the growth performance of weaned piglets [43]. Similarly, black tea extract, rich in polyphenols, seems to reduce growth performance [27], most likely by impacting diet palatability through its bitter and astringent taste. However, the reduction in feed intake and growth performance was not observed in this study. The antinutritive effects of tannins remain controversial. The present results are in accordance with a previous study that assessed the common antinutritive effects in pigs [44]. Although not statistically significant, the supplementation of HTE seems to improve feed intake and ADG in TA piglets compared to CO piglets. Other studies have confirmed the beneficial effects of tannins on feed efficiency, growth performance and concentration of beneficial *Lactobacillus* bacteria in weaned piglets [45] and broilers [46]. The antinutritive effect may be a question of dose and/or type of tannins.

The supplementation of 1% HTE in the diet reduced only the severity and duration of PWD and was not able to completely prevent the occurrence of PWD and to reduce ETEC shedding in the feces. This can be explained partly by the concentration of HTE in the chymus. Although not measured in this study, it has been suggested that tannins are already partly degraded by host or microbial enzymes when reaching the distal regions of the small intestine [47]. In general, hydrolysable tannins, such as the HTE used in the current study, are more susceptible to hydrolysis than condensed tannins [48]. As for other polyphenols, tannins are very reactive, and their effects seem not to be restricted to one type of molecule. Indeed, they can bind various constituents present in the gut, like iron or dietary proteins. For instance, the inhibition effect of HTE on *E*. *coli* toxins is suppressed when proteins are added to the medium [36]. The addition of iron in a medium partly reversed the positive effect of polyphenols on ETEC growth [27]. These components compete with microbial toxins or adhesins for tannins’ binding sites.

## Conclusion

The infectious model was repeatable, as the average fecal score, days in diarrhea, and percentage of piglets in diarrhea were within the same range in trials 1 and 2. Thus, the present infection model was suitable for studying approaches to preventing PWD using chestnut tannins. Adding 1% chestnut-tannin extract successfully decreased incidence and diarrhea severity but was not sufficient to reduce ETEC shedding. Increasing the dose of chestnut-tannin extract may improve the tannin efficiency, but care should be taken to stay within the “therapeutic window,” using a dose that does not induce antinutritional effects on protein digestion or feed palatability.
